# A Regional Multicenter Retrospective Analysis of Patients with Primary Central Nervous System Lymphoma Diagnosed from 2000-2012: Treatment Patterns and Clinical Outcomes

**DOI:** 10.7759/cureus.1512

**Published:** 2017-07-25

**Authors:** Eric C Burton, Beatrice Ugiliweneza, Murali K Kolikonda, Tanuj Saaraswat, Shiao Woo, Maxwell Boakye, Lennea Coombs, Renato LaRocca, Aaron Spalding

**Affiliations:** 1 Department of Neurology, University of Louisville; 2 Department of Neurosurgery, University of Louisville; 3 Radiation Oncology, University of Louisville; 4 Oncology, Norton Cancer Institute; 5 Radiation Oncology, Norton Cancer Institute

**Keywords:** primary cns lymphoma, methotrexate, radiation therapy

## Abstract

Introduction

Primary central nervous system lymphoma (PCNSL) is a rare tumor without a well-defined standard of care. For immunocompetent patients, therapeutic regimens have largely evolved from treatment with whole-brain radiation therapy (WBRT) to treating initially with systemic chemotherapy regimens that include high-dose (HD) methotrexate (MTX) with or without WBRT. Looking at population-based treatment trends may help define which therapies are most effective. This study was conducted to determine treatment patterns and outcomes for patients with PCNSL in the Louisville, KY metropolitan area during the period 2000 to 2012.

Methods

Data were collected by retrospective chart reviews of patients identified using the International Classification of Diseases (ICD) code from three major oncology practices in the Louisville metropolitan area during the period 2000 to 2012. Patients were excluded if they were under age 18, positive for human immunodeficiency virus (HIV), had histology other than B-cell lymphoma, or had systemic lymphoma.

Results

A total of 21 patients were identified. The median age was 65 years (range: 30 to 90). All patients were Caucasian, and the median Karnofsky performance status (KPS) score was 80 (range: 50 to 100). The ratio of males to females was 1:1.3. Median overall survival (OS) for all patients was 22 months (range: 1 to 155 months). Of 21 patients, 11 (52 percent) received chemotherapy regimens that included systemic HD-MTX at their initial diagnosis with a median OS of 22 months (range: 1 to 155 months). Nine of 21 patients (42 patients) were offered other therapies, including WBRT or non-MTX-based chemotherapies; they had a median OS of 5 months (range: 2 to 150 months). The median OS for patients receiving at least four cycles of HD-MTX was 40 months (range: 4 to 155 months).

Conclusions

This population-based study shows that patients with PCNSL and the ability to undergo HD-MTX-based therapy had a superior survival rate compared to those receiving radiation alone or other non-HD-MTX-based therapies.

## Introduction

Primary central nervous system lymphoma (PCNSL) is a rare tumor accounting for only two percent to three percent of all adult central nervous system (CNS) tumors in the United States [1]. The low incidence of PCNSL makes it difficult to do large, prospective, randomized trials. Given the rarity of these tumors, treatment recommendations are often based on small, single-institution Phase II studies rather than on historical controls. This is a major drawback in defining a standard of care and impedes the rational design of future clinical trials. It is not surprising that the best therapeutic strategy is still a subject of debate [2].

In the past 25 years, there has been a shift from using whole-brain radiation therapy (WBRT) for pre-radiation high-dose (HD) systemic methotrexate (MTX) regimens and delaying or declining WBRT altogether to avoid neurotoxicity [3]. Series reporting outcomes using HD-MTX regimens with or without radiotherapy (XRT) have shown increased median survival from approximately 12 months to 36 months compared to XRT alone [4-7]. However, questions remain. The potential benefit of consolidating XRT after chemotherapy is a matter of debate [2]. Some authors suggested chemotherapy alone as the main strategy. In addition, although systemic HD-MTX appears to be the most effective drug, the role of other systemic therapies is unclear. Better chemotherapy combinations and the optimal MTX dosage remain to be defined [3]. As a result, it would be of value to have a larger body of historical outcome data to inform future results and trial efforts.

The purpose of this study was to analyze a regional cohort of patients with PCNSL to assess treatment trends and outcomes in ordinary clinical practice. This may provide an important source of information for optimizing treatment in the future.

## Materials and methods

Study Cohort

We performed a retrospective review of the medical records of patients diagnosed with PCNSL and who were treated at three major oncology practices in the Louisville, Kentucky area from January 2000 to December 2012. The three institutions consisted of a university teaching hospital, a large metropolitan hospital, and a private practice oncology group. Patients were identified by International Classification of Diseases (ICD) code 9590/3. The date range was chosen because adequate medical records were available at all three institutions for that period.

Data Collection and Variables

The data collected included patient demographics, symptoms at presentation, the duration of the symptoms before diagnosis, age at diagnosis, the location of the tumor by imaging studies, staging results, Karnofsky performance score (KPS), cerebrospinal fluid (CSF) protein levels, serum lactate dehydrogenase (LDH) levels, treatments, initial progression-free survival (i.e., the time from diagnosis to relapse), survival after first recurrence, and overall survival (OS - the main outcome, i.e., the time from diagnosis to death). Our secondary outcomes were initial progression-free survival and the time from relapse to death. The institutional review boards (IRBs) of all three institutions approved this study.

Statistical Analysis

An analysis was performed on data from all 21 patients, and the effect of MTX treatment was evaluated by comparing treatment regimens containing MTX with treatment regimens without MTX.

The continuous variable (age) was summarized as a mean with standard deviation, median with associated interquartile range (IQR), and the range (minimum to maximum). Categorical variables (gender, race, type of surgery, and relapse rates) were summarized as a frequency count, with associated percentages. To evaluate whether the two treatment groups had statistically similar demographics, we compared age using the Wilcoxon rank sum test and categorical variables with the chi-square test.

Time-to-event variables (i.e., overall survival, time from diagnosis to relapse, and time from relapse to death) are presented with the median survival time and associated IQR as well as the full range (minimum survival months and maximum survival months). OS was compared between the two treatment groups using the log-rank test. A visualization of the survival curve is presented with Kaplan-Meier curves. 

Data pre-processing and analysis was performed in SAS 9.4 (SAS Inc., Cary, NC). All tests were two-sided with a significance level of 0.05.

## Results

A total of 21 patients were identified and included in the study (Table 1). The median age for all patients was 65 years (range: 30 to 90). All patients were Caucasian, and the median KPS at presentation was 80 (range: 50 to 100).

**Table 1:** Demography and clinical characteristics of patients in the study

Patient #	Age	Gender	Race	OS(M)	KPS	Protein	LDH
1	67	M	C	23	80	128	158
2	46	F	C	150			421
3	45	F	C	155	70	63	534
4	71	F	C	58			
5	66	F	C	1			138
6	65	F	C	5	70	76	245
7	43	F	C	4	100	30	
8	54	F	C	6	50	60	148
9	64	M	C	74	80		
10	65	M	C	27			
11	90	F	C				
12	72	M	C	5			
13	78	M	C	62			
14	64	F	C				
15	64	M	C				
16	71	M	C	5			
17	82	F	C	5			
18	30	M	C	19			
19	63	M	C	19			
20	72	F	C	2			
21	65	M	C				

OS(M): overall survival in months; KPS: Karnofsky performance score; LDH: lactate dehydrogenase; M: male; F: female; Protein: cerebrospinal fluid protein; C: Caucasian

The male-to-female ratio was 1:1.3, and the median length of time of presenting symptoms was 2 months (range: 0.25 to 12 months). Surgical data were available for 17 patients. A total of 11 patients underwent biopsy, 4 had a gross total resection (GTR), and 2 had subtotal resections. One of the patients included in the GTR group had ocular lymphoma and underwent a vitrectomy. This patient was observed without any other documented therapy. The median OS for all patients was 22 months (range: 1 to 155 months) (Table 2). 

**Table 2:** Patient characteristics and survival

				Initial Therapy		
Characteristics		All patients (n=21)		Without MTX (n=9)	With MTX (n=11)	p-value
Age						0.6201
	Mean (SD)	64.0 (13.8)		63.8 (18.3)	62.3 (9.48)	
	Median (IQR)	65.0 (63.0-71.0)		65.0 (54.0-72.0)	65.0 (63.0-67.0)	
	Range, min-max	30.0-90.0		30.0-90.0	43.0-72.0	
Gender, female, n (%)		12 (57.14)		5 (55.6)	7 (63.6)	0.7136
Race, white, n (%)		21 (100)		9 (100)	11 (100)	
Surgery						0.2505
	B, n (%)	11 (52.38)		4 (44.4)	7 (63.6)	
	GTR, n (%)	4 (19.05)		2 (22.2)	2 (18.2)	
	STR, n (%)	2 (9.52)		2 (22.2)	0 (0.00)	
	Missing, n (%)	4 (19.05)		1 (11.1)	2 (18.2)	
Relapsed, n (%)		9 (42.86)		2 (22.2)	7 (63.6)	
Outcome						0.0640
Survival months						0.7371
	Died/Total non-missing	11/17		4/6	7/10	
	Median (IQR)	22 (5-74)		5 (5-5)	22 (5-74)	0.6895
	Range, min - max	0-155*		0-150*	0-155*	
Months from relapse to death among those who relapsed before death						
	Died/Relapsed total	7/8				
	Median (IQR)	3 (1-19)				
	Range, min - max	0-98*				

Of the 21 patients (52 percent), 11 received systemic HD-MTX-based regimens (Table 3). The median age was 65 years (range: 43 to 72 years) and the median KPS for this group was 80 (range: 70 to 100). The median OS was 22 months (range: 0 to 155 months). Nine patients (42 percent) were offered WBRT alone and/or non-systemic MTX-based chemotherapy regimens. The median age for this group was 65 years (range: 30 to 90 years) and the median OS was 5 months (range: zero to 150 months). Only one patient’s KPS was recorded for this group (KPS = 50). One patient had no recorded treatment data available and was, consequently, not included in either treatment group.

**Table 3:** Patient treatment

Patient #	Sx(m)	Surgery	Initial Therapy	TR (Months)	Follow-Up Treatment	Tumor Location
1	2	B	6 HD-M/R/V/P	21	R/TMZ	D
2	1	B	Thalidomide			D
3	4	B	8 HD-M	39	W/R/TMZ	D
4	1	B	5 HD-M/P/V/C	47	HD-M/P/V/C	S
5	2	B	1 cycle HD-M			D
6	1.25	B	3 HD-M/C	1	W	S
7	10	B	7 HD-M/R/P/V-W	6	C	D
8	12	B	W			D
9	0.25	GTR	8 HD-M/C	47	M/R	S
10	0.5	GTR	1 HD-M-then W			S
11		B	W			S
12			RCHOP and 1 M			S
13						
14			10 HD-M			S
15			IT-M			
16		STR	RCHOP	3		S
17	5	GTR	W			D
18	3	STR	8 CVAD-C-IT-M			D
19		B	4 HD-M/C/R			S
20	1.5	B	2 Topotecan/IT-M			D
21		GTR	observation after vitrectomy			S

GTR: gross total resection; STR: subtotal resection; B: biopsy; TR: time to relapse; HD: high dose; M: methotrexate; R: rituximab; V: vincristine; T: topotecan; W: whole brain radiotherapy; IT: intrathecal chemotherapy; C: cytarabine; RCHOP: rituximab cyclophosphamide doxorubicin vincristine prednisone; CVAD: cyclophosphamide vincristine doxorubicin dexamethasone; TMZ: temozolomide; D: deep tumor location; S: superficial tumor location

Within the 21 patients included in this study, 14 different treatment regimens were used (Table 3). The 21 patients were divided into two cohorts: those that received systemic HD-MTX-based regimens (11 of 21 patients (52 percent)) and those that did not (9 of 21 patients (42 percent)). In the HD-MTX cohort, seven different treatment regimens were used. The most frequent regimen was single-agent HD-MTX given to three patients (27 percent). The second most-common regimen was HD-MTX combined with cytarabine in two patients (18 percent). In the nine patients that did not receive a systemic HD-MTX-based regimen, seven different treatments were used. The most frequent treatment was upfront WBRT given to three patients (33 percent).

Treatment information was available for six patients at the time of recurrence (Table 3). The treatments used were rituxan and temozolomide; WBRT, rituxan, and temozolomide; HD-MTX, procarbazine, vincristine, and cytarabine; WBRT; cytarabine; and HD-MTX and rituxan. The median survival time after recurrence was 7 months (range: 2 to 98 months).

We collected serum LDH and CSF protein data in order to give patients a prognostic score based on the international extranodal lymphoma study group where patients are stratified based on age, performance score, serum lactate dehydrogenase (LDH), cerebrospinal fluid (CSF) protein, and the involvement of deep regions of the brain (i.e., periventricular, basal ganglia, brainstem, and/or cerebellum). All variables were available for four patients in this cohort. Detailed data were not available for XRT or HD-MTX dosing.

## Discussion

Historically, treatment using WBRT for patients with PCNSL improved overall survival from 2 to 4 months to 12 to 15 months [8]. By the late 1980s and early 1990s, several small Phase II trials reported that treatment with systemic HD-MTX before WBRT for newly diagnosed patients improved survival to 33 to 42 months [6,9-10]. Additional confirmation of these early Phase II studies was provided by a large retrospective analysis published by Blay et al. in 1998, which examined a variety of chemotherapy regimens in 226 patients with PCNSL [11]. Blay et al. found that any regimen not including HD-MTX performed no better than WBRT alone [11]. However, with this combination of HD-MTX followed by WBRT, there was an accompanying increased risk of developing late neurotoxicity, particularly in older patients, which led to commonly deferring WBRT altogether [12]. Support for safely omitting upfront WBRT was provided in a prospective randomized Phase III study comparing patients treated using HD-MTX regimens with or without WBRT, where both arms showed similar overall survival (32.4 months vs. 36.1 months; hazard ratio (HR): 0.98 (95 percent); confidence interval (CI): 0.79 to 1.26; p = 0.98) [13-14].

In this study, we looked at a regional cohort of 21 PCNSL patients from the Louisville, KY metropolitan area that were diagnosed and treated from 2000 to 2012. We found that consistent with therapeutic recommendations after 2000, a slightly larger number of patients (11 of 21 or 52 percent) were treated with HD-MTX-based systemic chemotherapy regimens and in most cases, XRT was omitted or deferred.

Highlighted by this investigation is the lack of a standard of care even in newly diagnosed patients receiving HD-MTX-based regimens. Over the 13-year interval of this study, seven different upfront HD-MTX regimens were used in 11 patients. The two used most frequently were single agent HD-MTX (three cases) and HD-MTX in combination with cytarabine (two cases). The other regimens used in this patient cohort included HD-MTX, rituxan, vincristine, and procarbazine; HD-MTX, rituxan, vincristine, procarbazine, and WBRT; HD-MTX, vincristine, procarbazine, and cytarabine; HD-MTX, cytarabine, and rituxan; HD-MTX with rituxan and cyclophosphamide, doxorubicin, vincristine, and prednisone (R-CHOP).

In these 11 patients, upfront XRT was given only two times. In one case, WBRT was used for consolidation in a 43-year-old patient considered young enough to avoid neurotoxicity. In the second case, a patient was initially started on HD-MTX and then switched to WBRT because of toxicity from HD-MTX.

**Figure 1:**
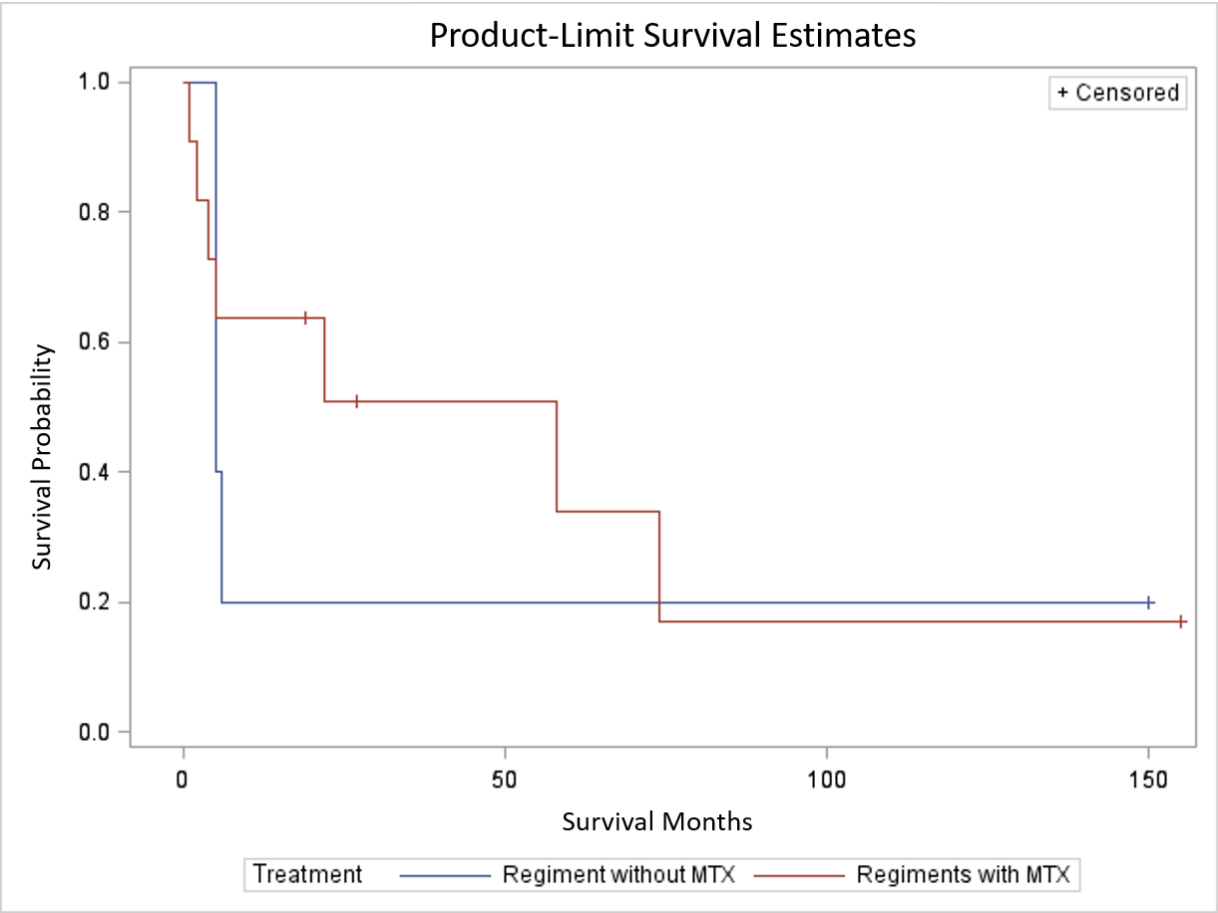
Survival estimates MTX: Methotrexate

Like previously published studies, we see that patients treated with HD-MTX regimens had a significantly improved survival compared to those patients that received alternative therapies (22 months vs. 5 months) (Figure 1) [11]. However, it remains unclear from our study how the initial treatment regimen was determined; HD-MTX as initial therapy may be a surrogate for high patient performance status (i.e., patients able to tolerate HD-MTX may do well regardless of initial therapy).

Many of the treatment regimens used on patients in this study reflect some of the combined modality trials for patients with PCNSL that were being conducted or were published during the interval of the study. In a Phase II study of 52 patients published in 2000 by Aubrey et al., patients were given HD-MTX plus procarbazine and vincristine and some patients received WBRT. The median overall survival for the 52 patients was 60 months [15]. However, the evaluation of this treatment approach in a larger multicenter Radiation Therapy Oncology Group (RTOG) trial with 102 patients demonstrated a median overall survival of only 36.9 months [16].

This regimen was later modified to include rituxan with reduced-dose WBRT followed by a high-dose cytarabine consolidation. The initial results of this multicenter single-arm Phase II trial published in 2007 were favorable, and, in 2013, the final results showed that survival on an intent-to-treat basis was 6.6 years in 52 patients [17-18].

Another chemotherapy combination reflected in this study was investigated in a Phase II randomized trial published by Ferreri et al. in 2009 [19]. This group evaluated HD-MTX-based induction with or without high-dose cytarabine followed by consolidative WBRT. They found the combination more effective with a three-year overall survival of 32 percent in the HD-MTX arm vs. 46 percent in the HD-MTX plus cytarabine arm (p = 0.07; HR: 0.65, 95 percent; CI: 0.38 to 1.13) [19].

One patient in this study group was treated with HD-MTX and R-CHOP, and although the combination chemotherapy regimen of R-CHOP is the best treatment for systemic non-Hodgkin’s lymphoma, it has not been shown to be effective for PCNSL [20]. Since 1994, several trials combining CHOP with cranial irradiation for PCNSL have been published, and none have demonstrated improved survival over WBRT alone or WBRT with HD-MTX [21-25]. More recently, the combination of HD-MTX and R-CHOP has been used to treat systemic non-Hodgkin’s lymphoma with CNS metastasis, but the presence of systemic disease should have excluded patients from this study [26].

In the nine patients that did not receive a systemic chemotherapy regimen that included HD-MTX, the most frequent alternative treatment was WBRT (three patients, 33 percent). The other alternative treatments were thalidomide; R-CHOP; intrathecal MTX (IT-MTX); topotecan with IT-MTX; and cyclophosphamide, vincristine, doxorubicin, and dexamethasone with IT-MTX. Although an explanation or justification for the use of alternative therapies is beyond the scope of this analysis, published studies describe the use of most of these agents in patients with PCNSL [27-28].

Information on treatment after recurrence was available for six patients. The treatments used at recurrence were rituxan and temozolomide; WBRT, rituxan, and temozolomide; HD-MTX, procarbazine, vincristine, and cytarabine; WBRT; cytarabine; and HD-MTX and rituxan.

Not surprisingly, treatments for recurrent disease were also varied. In this sample of patients, therapies used were already established as being effective in the upfront setting. In addition, temozolomide and rituxan to treat recurrent PCNSL has been looked at in several studies and can be used to treat recurrent disease in patients that cannot tolerate HD-MTX or have failed that therapy [29-30]. 

## Conclusions

Our study’s retrospective design along with incomplete records and a small sample size does not allow for statistically significant conclusions to be drawn with regard to treatments and outcomes. Additionally, selection bias does not allow rigorous unconfounded comparisons to be performed. However, this study does highlight the lack of a standard of care for patients with PCNSL at the community level and supports the use of HD-MTX-based regimens in the upfront treatment setting for these patients.

A large-population data-based study to help further the investigations of variability in treatment and mitigate selection bias issues would be valuable.
